# Muscle co-contraction in elderly people change due to postural stability during single-leg standing

**DOI:** 10.1186/s40101-017-0159-1

**Published:** 2017-12-16

**Authors:** Yoshitaka Iwamoto, Makoto Takahashi, Koichi Shinkoda

**Affiliations:** 1 Graduate School of Biomedical & Health Sciences, Hiroshima University, 2-3 Kasumi 1-chome, Minami-ku, Hiroshima, 734-8553 Japan; 2 Department of Biomechanics, Graduate School of Biomedical & Health Sciences, Hiroshima University, 2-3 Kasumi 1-chome, Minami-ku, Hiroshima, 734-8553 Japan; 3 Center for Advanced Practice and Research of Rehabilitation, Graduate School of Biomedical & Health Sciences, Hiroshima University, 2-3 Kasumi 1-chome, Minami-ku, Hiroshima, 734-8553 Japan

**Keywords:** Elderly people, Muscle co-contraction, Single-leg standing, Light touch, Center of pressure, Postural control

## Abstract

**Background:**

Muscle co-contraction is the simultaneous contraction of agonist and antagonist muscles crossing a joint, and it increases with age. This study primarily aimed to clarify the difference in the effect of a light fingertip contact to stationary surface on postural sway and muscle co-contraction during single-leg standing (SLS) between young and elderly groups; the secondary aim was to reveal the quantitative difference in the muscle co-contraction of the ankle joint among the three different support structure conditions in the elderly group.

**Methods:**

This study included eight young adults (age 23.4 ± 2.6 years) and nine community dwelling older adults (age 74.7 ± 3.4 years). The task was SLS under the following conditions: (1) no supporting structure, FR; (2) light index fingertip contact to a stationary supporting structure (to touch in force < 1 N), LT; and (3) dependence on a supporting structure for stabilization as desired, DO. Center of pressure (COP) variables [root-mean-square distance (RDIST), total excursion (TOTEX), mean velocity (MVELO), and standard deviation area (AREA-SD)] and the co-contraction index (CI) between the tibialis anterior and soleus were measured using surface electromyography.

**Results:**

With regard to the effect of the light fingertip contact to stationary surface, in the young group, TOTEX, MVELO, AREA-SD, and CI during SLS were smaller under the LT condition than under the FR condition. However, in the elderly group, only AREA-SD and CI were smaller under the LT condition than under the FR condition. No significant difference was observed in COP variables and CI under the DO condition between the young and elderly groups.

**Conclusion:**

Both young and elderly groups could decrease muscle co-contraction using the light fingertip contact. On the other hand, in the elderly group, COP variables showed a limited effect from the light fingertip contact; only the “sway” domain measure (AREA-SD). Both young and elderly groups showed the smallest CI under the DO condition. Therefore, the elderly group could decrease muscle co-contraction of the ankle joint depending on postural stability.

## Background

Muscle co-contraction is the simultaneous contraction of agonist and antagonist muscles crossing a joint [1]. Several studies have shown larger lower limb muscle co-contraction in elderly people than in young people during static and dynamic postural control [2–5]. Larger muscle co-contraction in elderly people has often been described as a compensatory strategy to enhance postural stability, with stiffening of their joints [2, 3]. In contrast, some studies have reported that a rigid posture induced by strong muscle co-contraction reduces flexibility of postural control [6, 7]. However, it was unknown whether the status of muscle co-contraction of the ankle joint changes depending on the stability in each task in elderly people. Therefore, investigating the amount of muscle co-contraction during several postural control tasks that involve different stabilities is necessary to clarify whether the elderly people could modulate their muscle co-contraction depending on their stability during the tasks.

The assistive devices could improve postural stability by providing mechanical advantages [8]. In addition to the mechanical advantage, additional haptic sensory input through a fingertip decreased postural sway during bipedal standing [9–11]. Jeka and Lackner [9] showed that touching a fingertip to a stationary surface at a force far below that adequate for physical support (< 1 N) can enhance the perception of body orientation and stabilize postural control during bipedal standing in young people. This type of effects by the light fingertip contact have also been observed in elderly people in another study [12]. Bipedal standing is a fundamental task to cope with activities of daily life. Moreover, maintaining single-leg standing (SLS) is more challenging than maintaining bipedal standing, and SLS duration is correlated with the incidence of falls in elderly people [13, 14]. Therefore, it is important to study about the effect of the light fingertip contact on postural sway during not only bipedal standing but also SLS in the elderly people.

Haptic sensory information can affect not only postural sway but also muscle activation. Jeka and Lackner [15] showed that the light fingertip contact to stationary surface leads to decreased levels of EMG activity of the lower limb during bipedal standing in young people. Moreover, a light gripping cane leads to decreased muscle co-contraction during SLS in young people [16]. These findings demonstrated the possibility the haptic sensory information with the light fingertip contact might decrease muscle co-contraction of the ankle joint during SLS even in elderly people. However, no study has evaluated muscle co-contraction of the ankle joint during SLS in elderly people. We hypothesized that the postural stability with the assistive device and the light fingertip contact could change the state of muscle co-contraction in elderly people.

In our study, we measured the center of pressure (COP) and muscle co-contraction of the ankle joint using the force plates and surface electromyography (EMG) during SLS in two different age groups (young and elderly groups). In addition, we prepared three different support structure conditions for regulating postural stability (no supporting structure, light index fingertip contact to a stationary surface, and dependence on a supporting structure for stabilization as desired). This study primarily aimed to clarify the difference in the effect of the light fingertip contact on postural sway and muscle co-contraction during SLS between young and elderly people; the secondary aim was to reveal the quantitative difference in muscle co-contraction of the ankle joint among the three support structure conditions in the elderly group.

## Methods

### Participants

This study included eight young adults (five males and three females; mean age 23.4 ± 2.6 years; mean height 1.63 ± 0.10 m; mean body mass 59.8 ± 14.0 kg) and nine community-dwelling healthy older adults (five males and four females; mean age 74.7 ± 3.4 years; mean height 1.57 ± 0.10 m; mean body mass, 57.9 ± 7.1 kg).

Participants were excluded if they had neurological impairment, severe cardiovascular disease, persistent joint pain, or musculoskeletal impairment. Each participant identified their dominant leg as the leg they considered stronger and the leg they used to kick a ball [17]. In all participants, the right leg was the dominant leg. Participants provided written informed consent to participate in this study after receiving a detailed explanation regarding the purpose, potential benefits, and risks involved with participation. The experimental procedures of the study were conducted according to the Declaration of Helsinki and were approved by the Ethical Committee for Epidemiology of Hiroshima University (approval number E-467).

### Task

The task involved SLS on two force plates (each leg on each force plate) (TF-4060-A, Tec Gihan, Kyoto, Japan) with the dominant leg and with eyes open.

The participants were instructed to stand barefoot (each foot on a separate force plate) with their hands hanging relaxed along the side of the trunk and to look straight ahead. They were first asked to stand relaxed with the weight evenly distributed between both feet for at least 1 s and were then verbally instructed to lift the non-dominant leg. They tried to remain standing on dominant leg as long and steady as possible for up to 20 s. We measured 15 s of SLS, excluding the beginning and ending of the movements. In each SLS task, a 3-s period in the middle of the task was selected to calculate each parameter.

**Fig. 1:**
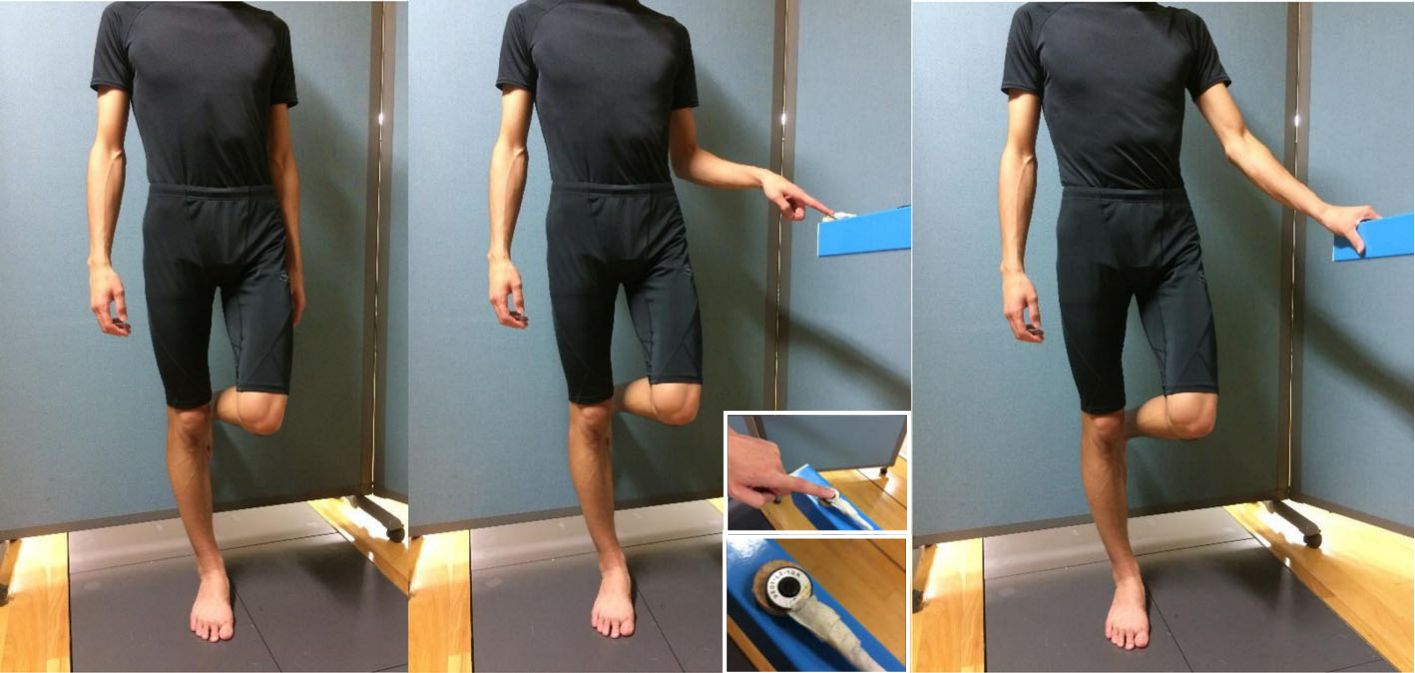
Schematic depiction of the test conditions. Participants stood with their dominant leg on the force plate under the following three conditions: (1) no supporting structure, FR (left); (2) light index fingertip contact to a stationary surface (to touch in force < 1 N), LT (middle); and (3) dependence on a supporting structure for stabilization as desired, DO (right)

The participants performed the task under the following three conditions (Fig. 1): (1) no supporting structure, FR; (2) light index fingertip contact to a stationary surface (to touch in force < 1 N), LT; and (3) dependence on a supporting structure for stabilization as desired, DO. Under each condition, participants performed several practice trials before the actual measurement. Moreover, we monitored whether the index finger applied > 1 N to the supporting structure using a pressure sensor (9E01-2L-10k, NEC-Sanei, Tokyo, Japan). If index finger’s pressure exceeded 1 N, the trial was performed again. In LT and DO conditions, participants were asked to apply the support before raising their leg.

### Postural sway measurement

We measured the ground reaction force with a force plate using the Nexus 2 software (Vicon Motion Systems, Oxford, UK) at 1000 Hz, and the collected data was then low-pass filtered with a fourth-order Butterworth filter at a cutoff frequency of 20 Hz. We calculated the coordinates of COP at a sampling frequency of 100 Hz with Nexus 2.

From the coordinates of COP, we calculated the following variables to evaluate postural sway during the task: root-mean-square distance (RDIST), total excursion (TOTEX), mean velocity (MVELO), and standard deviation area (AREA-SD) [18, 19].

### Muscle activation measurement

EMG data were collected using the Multichannel Amplifier (MEG-6108, Nihon Kohden, Tokyo, Japan) at a sampling frequency of 1000 Hz. The skin of the right leg over the fibular head, tibialis anterior (TA), and soleus (SOL) muscles were shaved and then cleaned with a skin preprocessing agent (Skin Pure, Nihon Kohden, Tokyo, Japan). Active electrodes (NM-512G, Nihon Kohden, Tokyo, Japan) were placed in line with the muscle fibers [20]. The ground electrode was affixed to the skin over the fibular head.

EMG activity was recorded from the TA and SOL while the participants were performing maximal voluntary contraction (MVC). MVC of the SOL was obtained during maximal isometric plantar flexion with standing calf raise, and that of the TA was recorded during maximal isometric dorsiflexion of the ankle at 90° (anatomically neutral position). Participants were asked to perform each MVC twice for 5 s, with a 30-s pause between the tasks. Strong verbal encouragement was provided during every contraction to promote maximal effort [21].

The original raw EMG signal was band-pass filtered at a range of 20–500 Hz. Then, we computed the root-mean-square amplitude of the signal using a 50-ms window [21]. EMG of each muscle was then normalized with the EMG value during MVC.

To evaluate the relative level of co-contraction of the TA and SOL muscles, the co-contraction index (CI) was calculated using the method described by Falconer and Winter [22]. We introduced the detailed explanation in our previous study [23]. We used following equations:

1 $$ \mathrm{CI}\left[\%\right]=\frac{2{I}_{\mathrm{ant}}}{I_{\mathrm{total}}}\times 100 $$

where *I*_ant_ is the total antagonistic activity, calculated with the following Eq. (2)

2 $$ {I}_{\mathrm{ant}}={\int}_{t_1}^{t_2}{\mathrm{EMG}}_{\mathrm{TA}}(t)\mathrm{dt}+{\int}_{t_2}^{t_3}{\mathrm{EMG}}_{\mathrm{SOL}}(t)d $$

where *t*_1_ to *t*_2_ is the period TA was decided as the antagonist, *t*_2_ to *t*_3_ is the period SOL was decided as the antagonist, and *I*_total_ is the total muscle activity while participants performed the SLS, calculated using the following Eq. (3):

3 $$ {I}_{\mathrm{total}}={\int}_{t_1}^{t_3}\left[{\mathrm{EMG}}_{\mathrm{TA}}+{\mathrm{EMG}}_{\mathrm{SOL}}\right](t)\mathrm{dt} $$

### Statistical analysis

We first used Shapiro–Wilk test to assess all variables for normality. Then, if the variables were according to the normal distribution, we used Student’s *t* test. Otherwise, we used Wilcoxon or Mann–Whitney test. A sequential Bonferroni correction factor was used to adjust the alpha level for multiple comparisons [24]. Data were statistically analyzed using the SPSS software (Windows version 22, IBM Japan, Tokyo, Japan). The significance level for all tests was 5%.

## Results

We demonstrated results of the COP variables in Table 1. With regard to age-associated differences in COP variables, AREA-SD was significantly larger in the elderly group than in the young group under the FR condition (*p* = 0.008). Moreover, under the LT condition, RDIST, and AREA-SD were larger in the elderly group than in the young group (*p* = 0.005 and 0.001, respectively). In contrast, under the DO condition, no significant difference was observed between the two age groups (RDIST, TOTEX, MVELO, and AREA-SD, *p* = 0.35, 0.50, 0.50, and 1, respectively).

**Table 1:** COP variables of two age groups’ during single-leg standing in each condition

	Young			Elderly		
	FR	LT	DO	FR	LT	DO
RDIST [mm]	6.04 ± 2.66	3.73 ± 1.08	2.65 ± 0.64^‡‡‡^	6.71 ± 3.06	6.10 ± 1.75^***^	3.14 ± 1.36^‡‡‡§§§^
TOTEX [mm]	271.31 ± 53.16	255.41 ± 49.76^†††^	254.51 ± 57.51^‡‡^	315.64 ± 26.09	302.89 ± 35.35	271.91 ± 44.10^‡‡‡§§§^
MVELO [mm/s]	90.44 ± 17.72	85.14 ± 16.59^†††^	84.84 ± 19.17^‡‡^	105.21 ± 8.70	100.96 ± 11.78	90.64 ± 14.70^‡‡‡§§§^
AREA-SD [mm^2^]	53.27 (40.14–82.24)	30.45 (28.28–33.32)^†^	11.34 (6.18–16.72)^‡‡‡§§§^	108.69 (88.75–133.77)^***^	73.59 (56.74–80.91)^***†††^	7.96 (7.18–12.96)^‡‡‡§§§^

Data are mean ± standard deviation or median (interquartile range)

*RDIST* the root-mean-square (RMS) distance of COP, *TOTEX* the total excursion of COP, *MVELO* the mean velocity of COP, *AREA-SD* standard deviation area of COP, *FR* no supporting structure, *LT* light touch of the index finger to a supporting structure (to touch in force < 1 N), *DO* dependence on a supporting structure for stabilization as desired]

Significant differences between the young and elderly groups are indicated by asterisks (***p < 0.017). Significant differences between the FR and LT conditions are indicated by daggers (^†^ *p* < 0.05, ^†††^ *p* < 0.017). Significant differences between the FR and DO conditions are indicated by double daggers (^‡‡^ *p* < 0.025, ^‡‡‡^ *p* < 0.017). Significant differences between the LT and DO conditions are indicated by section signs (^§§§^ *p* < 0.017)

In the young group, TOTEX, MVELO, and AREA-SD were smaller under the LT condition than under the FR condition (*p* = 0.014, 0.014, and 0.049, respectively). However, in the elderly group, only AREA-SD was significantly smaller under the LT condition than under the FR condition (*p* = 0.011).

In contrast, in the young group, AREA-SD was smaller under the DO condition than under the LT condition (*p* = 0.017), and in the elderly group, RDIST, TOTEX, MVELO, and AREA-SD were smaller under the DO condition than under the LT condition (*p* = 0.003, 0.006, 0.006, and 0.008, respectively). Both the age groups showed smaller value for COP variables under the DO condition than under the FR condition (the young group: *p* = 0.012, 0.022, 0.022, and 0.012; the elderly group: *p* = 0.007, 0.006, 0.006, and 0.008, respectively).

**Fig. 2:**
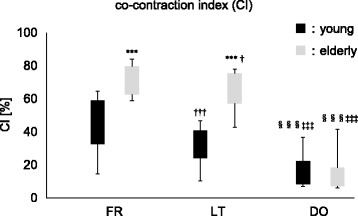
Comparison of the co-contraction index (CI). Significant differences between the young and elderly groups are indicated by asterisks (^*^ *p* < 0.05, ^**^ *p* < 0.025, ^***^ *p* < 0.017). Significant differences between the FR and LT conditions are indicated by daggers (^†^ *p* < 0.05, ^††^ *p* < 0.025, ^†††^ *p* < 0.017). Significant differences between the FR and DO conditions are indicated by double daggers (^‡^ *p* < 0.05, ^‡‡^ *p* < 0.025, ^‡‡‡^ *p* < 0.017). Significant differences between the LT and DO conditions are indicated by section signs (^§^ *p* < 0.05, ^§§^ *p* < 0.025, ^§§§^ *p* < 0.017). [FR, no supporting structure; LT, light touch of the index finger to a supporting structure (to touch in force < 1 N); DO, dependence on a supporting structure for stabilization as desired]

Figure 2 shows CI of the ankle joint (TA-SOL) between the two age groups during SLS under each condition. CI was significantly larger in the elderly group than in the young group under FR and LT conditions (*p* < 0.01 and 0.01, respectively), where no statistical difference was observed in the DO condition between these groups (*p* = 0.67). Moreover, in both the age groups, CI was significantly smaller under the LT condition than under the FR condition (the young group *p* = 0.012, the elderly group *p* = 0.028) and was significantly larger under the LT condition than under the DO condition (the young group *p* = 0.012, the elderly group *p* = 0.008).

## Discussion

In this study, we measured postural sway and muscle co-contraction of the ankle joint during SLS in the young and elderly group under three different support conditions (FR, LT, and DO). We hypothesized that the postural stability with the assistive device and the light fingertip contact could change the state of muscle co-contraction in elderly people. Our results showed that in the elderly group, the light fingertip contact had a different effect on postural sway compared with that in the young group. Conversely, with regard to muscle activation, both groups showed decreased muscle co-contraction of the ankle joint with the assistive device and the light fingertip contact to the stationary surface. Both groups could control the amount of muscle co-contraction of the ankle joint depending on external postural stability.

We asked them to stand with their dominant leg. In general, the leg with which people kick a ball is considered as the dominant leg, and the other leg is considered as the support leg. There might be functional differences between the dominant and support legs; however, some studies have shown that there is no difference between the two legs in postural control during SLS [25, 26]. Bohannon et al. showed no differences about the duration to keep SLS between on right and left legs among 20–80-year-old subjects [25]. Moreover, Hoffman et al. showed no difference about sway area and sway path length of COP between the functionally dominant and non-dominant lower limbs during SLS [26]. Therefore, we could adapt our results on the non-dominant leg as well.

Aging is associated with the deterioration of standing balance [27]. Moreover, maintaining SLS is more challenging for elderly people [28]. In this study, AREA-SD was larger in the elderly group than in the young group under the FR condition, and this finding is in agreement with previous results [29]. AREA-SD was calculated by multiplying *π* and standard deviation in each direction (anteroposterior and mediolateral) [19]. Thus, this variable indicated the degree of sway. The results showed that compared with the young group, the elderly group controlled their COP more unstably in the narrow base of support (BOS) during SLS.

In this study, with regard to postural sway parameters, the young group could decrease TOTEX, MVELO, and AREA-SD during SLS with the light fingertip contact to a stationary surface. However, the elderly group could decrease only AREA-SD during SLS with the light fingertip contact. TOTEX and MVELO are considered time-domain “distance” measures [18], whereas AREA-SD is considered a “sway” measure [19]. The light fingertip contact to a stationary surface can reduce postural sway during bipedal standing in elderly people [12] as well as in young people [9, 11, 30–32]. Moreover, Holden et al. [10] showed the effect of light touch on COP parameters during SLS in young subjects. However, maintaining SLS was a more challenging task for elderly people because of a smaller BOS and elderly people generally tend to extend their BOS in response to an overall decline in postural stability [33]. The light fingertip contact was not a mechanical support and could not extend BOS. Therefore, the effect of the light fingertip contact was limited in the elderly group compared with that in the young group in this study.

Elderly group demonstrated larger muscle co-contraction of the ankle joint (TA-SOL) than young group during SLS under the FR condition (Fig. 2). Previous studies showed greater lower limb muscle co-contraction during static and dynamic postural control tasks (quiet standing, functional reach, and gait) in elderly people than in young people [2–5]. In our study, we selected SLS as the task. SLS was discriminated as a static postural control, although it was more unstable than bipedal standing because of narrow BOS. To date, no study has compared muscle co-contraction during such an unstable SLS between young and elderly people. The amount of muscle co-contraction of the ankle joint could be attributed to age difference, even in keeping SLS, which was more unstable than bipedal standing. It indicated that there was a critical aging effect on muscle co-contraction in the elderly group. Jeka and Lackner [15] showed a reducing effect of the light fingertip contact to a stationary surface on postural sway and muscle activity during bipedal tandem stance. Moreover, Ohsihita and Yano [16] measured EMG of TA and gastrocnemius during SLS with light gripping of a cane according to the previous report that a light touch effect is also observed for light grasping of an unstable object [34]. The authors then calculated CI based on the study by Rudolph et al. [35] and showed a smaller CI with the lightly gripping of the cane during SLS than without the cane. However, no study has clarified whether haptic information by the light fingertip contact could decrease lower muscle co-contraction during SLS in elderly and young people. In our study, CI decreased in the young and elderly groups with the light fingertip contact. In general, elderly people tend to increase muscle co-contraction to stiffen the ankle joint as a postural strategy. However, our findings indicated that the elderly group could use haptic information from the light fingertip contact to a support surface to reduce excessive muscle co-contraction of the ankle joint. These findings might be applicable for elderly people to practice maintaining balance more effectively. This kind of balance training could potentially lead to more efficient postural control strategy with decreasing muscle co-contraction to prevent falls among elderly people.

Under the DO condition, there were no significant differences between young and elderly group with regard to COP variables and CI. Moreover, both the age groups demonstrated the smallest COP variables and CI under the DO condition. When people grasp a handrail during upright standing, they can increase BOS, which allows them to generate forces at the hand to counteract perturbations [36]. In our study, young and elderly groups showed decreased limb muscle co-contraction when tightly grasping a supporting device. Elderly group could decrease co-contraction, even during SLS, with stable postural control associated with an enlarged BOS.

In our study, the changes in muscle co-contraction of the ankle joint during SLS under each condition were similar between the elderly and young group. In other words, the association with regard to CI was similar among the three support conditions (the largest was for FR followed by LT and DO) in both the age groups. Although the elderly group might have had some aging effects, both age groups could decrease muscle co-contraction, which was very interesting. Elderly people have a possibility to modulate the amount of muscle co-contraction depending on the external environment. Adapting to various situations in exercise could make a margin to control a sudden unknown perturbation for elderly people.

The small sample size might be considered a limitation of our study. However, we found several statistically significant between-condition and between-group differences. Therefore, we believed our results well reflected the characteristics of both age groups and our sample size was adequate to discuss age differences and the effect of postural stability on muscle co-contraction during SLS.

## Conclusions

Both young and elderly groups could decrease muscle co-contraction with the light fingertip contact. On the other hand, the elderly group showed a limited effect on COP variables from light fingertip contact, only the sway domain measure (AREA-SD). Both the age groups showed the smallest CI under the DO condition. Therefore, the elderly group could decrease muscle co-contraction of the ankle joint depending on postural stability.

## Abbreviations

AREA-SD: Standard deviation area
BOS: Base of support
CI: Co-contraction index
COP: Center of pressure
DO: Dependence on supporting structure
EMG: Electromyography
FR: No supporting structure
LT: Light touch of the index finger to a supporting structure
MVC: Maximal voluntary contraction
MVELO: Mean velocity
RDIST: Root-mean-square distance
SLS: Single-leg standing
SOL: Soleus
TA: Tibialis anterior
TOTEX: Total excursion
